# Optimal reactive nitrogen control pathways identified for cost-effective PM_2.5_ mitigation in Europe

**DOI:** 10.1038/s41467-023-39900-9

**Published:** 2023-07-17

**Authors:** Zehui Liu, Harald E. Rieder, Christian Schmidt, Monika Mayer, Yixin Guo, Wilfried Winiwarter, Lin Zhang

**Affiliations:** 1 Laboratory for Climate and Ocean-Atmosphere Studies, Department of Atmospheric and Oceanic Sciences, School of Physics, Peking University, Beijing, 100871 China; 2 International Institute for Applied Systems Analysis (IIASA), A-2361 Laxenburg, Austria; 3 Institute of Meteorology and Climatology, Department of Water, Atmosphere and Environment, University of Natural Resources and Life Sciences (BOKU), A-1180 Vienna, Austria; 4 Institute of Environmental Engineering, University of Zielona Góra, PL 65-417 Zielona Góra, Poland

**Keywords:** Atmospheric chemistry, Atmospheric chemistry, Environmental impact

## Abstract

Excess reactive nitrogen (Nr), including nitrogen oxides (NO_x_) and ammonia (NH_3_), contributes strongly to fine particulate matter (PM_2.5_) air pollution in Europe, posing challenges to public health. Designing cost-effective Nr control roadmaps for PM_2.5_ mitigation requires considering both mitigation efficiencies and implementation costs. Here we identify optimal Nr control pathways for Europe by integrating emission estimations, air quality modeling, exposure-mortality modeling, Nr control experiments and cost data. We find that phasing out Nr emissions would reduce PM_2.5_ by 2.3 ± 1.2 μg·m^−3^ in Europe, helping many locations achieve the World Health Organization (WHO) guidelines and reducing PM_2.5_-related premature deaths by almost 100 thousand in 2015. Low-ambition NH_3_ controls have similar PM_2.5_ mitigation efficiencies as NO_x_ in Eastern Europe, but are less effective in Western Europe until reductions exceed 40%. The efficiency for NH_3_ controls increases at high-ambition reductions while NO_x_ slightly decreases. When costs are considered, strategies for both regions uniformly shift in favor of NH_3_ controls, as NH_3_ controls up to 50% remain 5-11 times more cost-effective than NO_x_ per unit PM_2.5_ reduction, emphasizing the priority of NH_3_ control policies for Europe.

## Abstract (web summary)

Reactive nitrogen (Nr) contributes strongly to PM_2.5_ air pollution in Europe. Here, authors identify diverse Nr control pathways for Europe depending on emission and pollution formation and a priority of NH_3_ control when costs are considered.

## Introduction

Ambient PM_2.5_ (fine particulate matter with an aerodynamic diameter ≤2.5 µm) air pollution is one of the leading risk factors for premature mortalities worldwide, according to the Global Burden of Disease (GBD) study^1^, responsible for millions of deaths and lost years of healthy life annually in recent years^1–3^. Long-term policies for PM_2.5_ mitigation have been implemented in many countries and have effectively reduced PM_2.5_ concentrations^4–7^. However, large numbers of people are still exposed to harmful PM_2.5_ levels even in places with relatively clean ambient air such as Europe^7^; 59% of European stations exceed the World Health Organization (WHO) guideline for the PM_2.5_ annual mean (10 μg·m^−3^) in 2019^8^. Recent epidemiological studies also demonstrated that PM_2.5_ air pollution can affect human health at very low levels^9,10^. The WHO thus released an updated guideline value for PM_2.5_ annual mean concentrations (5 μg·m^−3^)^3^, which was exceeded at 97% of European monitoring stations in 2019^8^. This poses a tremendous challenge for cleaning up European air as much more stringent mitigation measures will be needed to achieve such an ambitious goal. The WHO also suggested interim targets to be considered, although recognizing that interim targets are insufficient to remove adverse health impacts.

Excess reactive nitrogen (Nr), including nitrogen oxides (NO_x_ = NO + NO_2_), ammonia (NH_3_), nitrate (NO_3_^−^), and ammonium (NH_4_^+^) are recognized environmental threats to ecosystems, deteriorating the quality of air, soil, and water^11,12^. Anthropogenic Nr sources have dramatically increased since 1960^13^, exacerbating the global nitrogen cycle and consequent damaging effects on human health and ecosystems^13,14^. Capping anthropogenic Nr emissions (mainly NO_x_ and NH_3_) is a high priority for environmental protection^15,16^. In particular, Nr controls benefit PM_2.5_ mitigation because both NO_x_ and NH_3_ are precursors of secondary inorganic aerosols (SIAs, including sulfate, nitrate, and ammonium) components in PM_2.5_, apart from sulfur dioxide (SO_2_)^14,17,18^. SIAs strongly contribute to the PM_2.5_ mass concentrations in Europe^19–21^, contributing above 50% of total annual PM_2.5_ mass concentrations in parts of Europe, i.e., Germany, the Netherlands, and Belgium^22–24^. Atmospheric abundance of NH_3_ and NO_x_ gases determine the formation of SIAs, and effectiveness of PM_2.5_ mitigation from Nr controls^25–27^. NH_3_ preferably reacts with sulfuric acid (H_2_SO_4_, produced by the oxidation of SO_2_) to form ammonium sulfate aerosol, and with more NH_3_ available, further reacts with nitric acid (HNO_3_, produced by the oxidation of NO_x_) to form ammonium nitrate aerosol.

The revised Gothenburg Protocol has set national Nr emission ceilings for 2020, i.e., 42% NO_x_ emission reductions and 6% NH_3_ emission reductions in 2020 relative to 2005 for the European Union (EU)^28^ and other participating countries. The National Emissions Ceiling Directive further establishes national Nr emission reduction targets in 2030, i.e., 63% NO_x_ emission reductions and 19% NH_3_ emission reductions in 2030 relative to 2005 in the EU^29^. All existing national targets show more ambitious controls for NO_x_ than NH_3_. Most countries have not prioritized limiting NH_3_ emissions in part due to uncertainties in NH_3_ sources and concerns about its control effectiveness for PM_2.5_ mitigation, in addition to food security concerns^17^, with agriculture being the dominant source of NH_3_. However, recent studies found agricultural (mainly NH_3_) emissions make the largest relative contribution to PM_2.5_ mortality in Europe among all sources^2,30,31^. Gu et al. ^18^ also found that the cost of 50% NH_3_ emissions abatement is much less than that of NO_x_ emissions globally. However, the priority for NO_x_ or NH_3_ emission reductions to meet the updated WHO guideline and zero pollution action plan^32^ in Europe remains uncertain.

In this study, we quantify the contribution and efficiency of Nr emission reductions for PM_2.5_ mitigation in Europe for 2015 and derive the optimal pathway for Nr emission controls. We use recent European emission estimates, a regional air quality model, the newly developed exposure mortality model, Nr control scenarios, and emission control costs to systematically analyse the impact of Nr emission controls on PM_2.5_ air pollution (Methods). We demonstrate that Nr emission controls can reduce PM_2.5_ concentrations, PM_2.5_-related health impacts, and help achieve the WHO guideline in Europe. The optimal pathway targeting PM_2.5_ abatement changes towards prioritizing NH_3_ measures after considering control costs, indicating NH_3_ emission reductions are the most cost-effective way to combat European PM_2.5_ air pollution.

## Results and discussion

### The contribution of Nr emissions on PM_2.5_ air pollution

Our ECLIPSE inventory derived from the GAINS (Greenhouse gas and Air pollution Interactions and Synergies) model (Methods) estimates the total anthropogenic NO_2_ and NH_3_ emissions over Europe in 2015 to be 3.7 Tg N and 4.4 Tg N, respectively, which are comparable to other emission inventories (Supplementary Table 1). Considering monthly time factors, NH_3_ emissions tend to peak during the warm season (April-September), while NO_x_ emissions peak during the cold season in Europe. Such seasonality appears stronger in our estimates than in the EDGAR and EMEP inventories (Supplementary Fig. 1). Both NH_3_ and NO_x_ emissions are higher in the western part of Europe than in the east. This ECLIPSE emissions inventory is used as an input to the Weather Research and Forecasting model coupled with Chemistry (WRF-Chem) regional air quality model to assess the impacts of Nr emission reductions on PM_2.5_ air pollution in Europe. A series of WRF-Chem simulations are conducted over Europe for the representative months (January, April, July, and October) in 2015 (Methods). The baseline simulation in Europe, after improving simulated organic carbon (OC) and dust by matching observations of PM_2.5_ components (Supplementary Figs 2 and 3), well captures measured surface PM_2.5_ concentrations with the correlation coefficients (*R*) > 0.59 and mean bias (MB) < −6% (Supplementary Fig. 3). The magnitudes and variations of the observed SIAs concentrations are generally captured by the baseline simulation, except in summer, when the model underestimates nighttime nitrate volatility and overestimates nitrate concentrations. The simulated surface annual NH_3_ concentrations are also in good agreement with measurements in Europe with R = 0.92 and MB of within −3% (Supplementary Fig. 4).

**Fig. 1:**
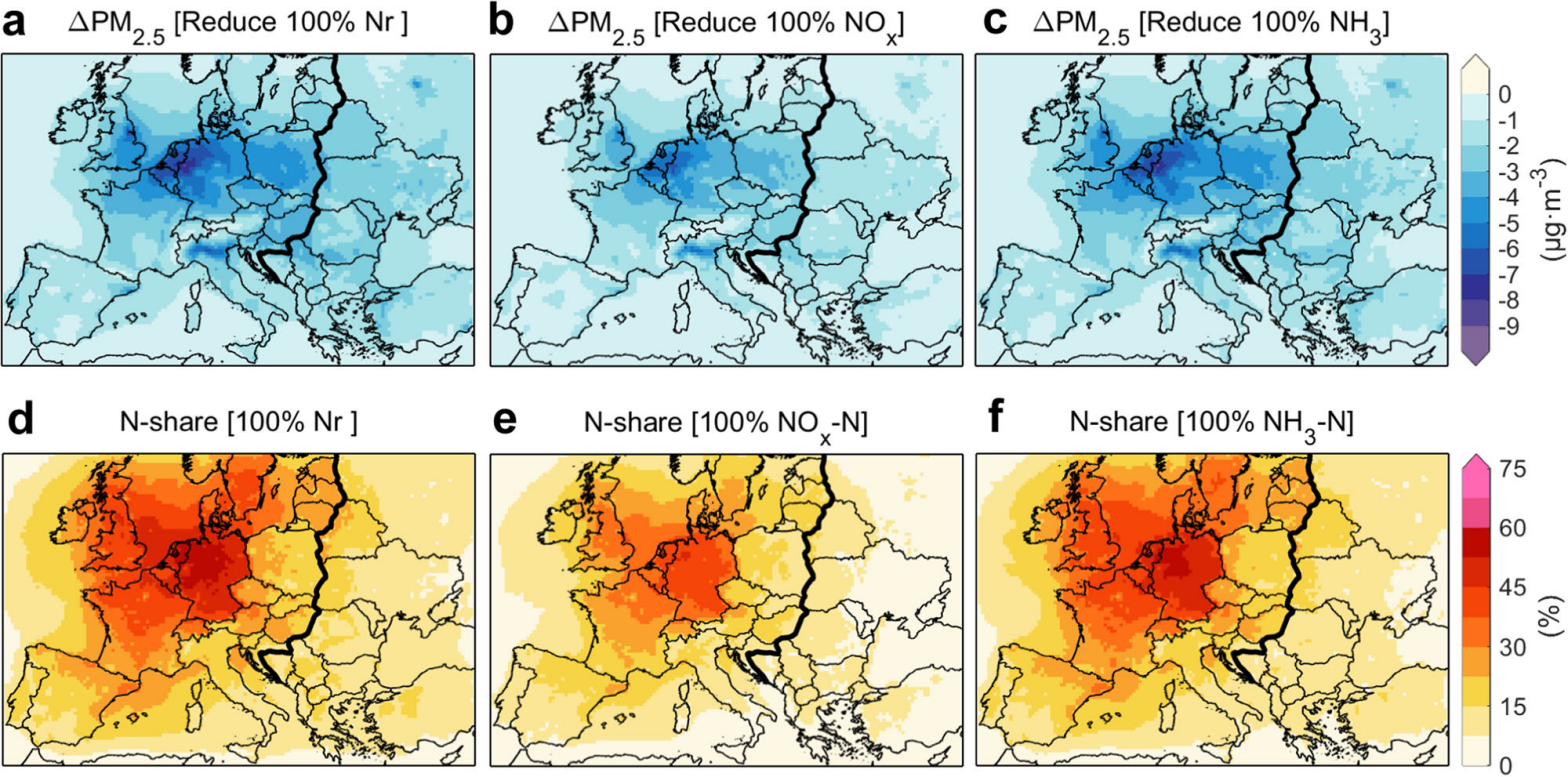
Contribution of reactive nitrogen (Nr) emissions to PM_2.5_ air pollution over Europe in 2015. **a**–**c** Changes of PM_2.5_ concentrations induced by phasing out anthropogenic Nr (NO_x_ + NH_3_) emissions (**a**), NO_x_ emissions (**b**), and NH_3_ emissions (**c**) respectively. **d**–**f** N-shares of PM_2.5_ air pollution associated with Nr (**d**), NO_x_ (**e**), and NH_3_ (**f**) emissions.

The contribution of anthropogenic Nr emissions to PM_2.5_ air pollution can be calculated as the difference between the baseline simulation and a sensitivity simulation with anthropogenic Nr emissions set to zero (Methods). Figure 1 shows that the reduction in regional annual mean PM_2.5_ concentrations when phasing out anthropogenic Nr emissions is 2.3 ± 1.2 μg·m^−3^ (mean ± standard deviation) in Europe for 2015. The response to such emission controls for PM_2.5_ concentrations is stronger in Western Europe than in Eastern Europe, with the largest effects occurring in the Netherlands, Belgium and northern Germany. For evaluation, we separate Europe into Western Europe and Eastern Europe along country borders, guided by the spatial difference of PM_2.5_ changes from Nr emission controls (the thick black line in Fig. 1). We further apply a metric of N-share^18^ to quantify the contribution of Nr compounds to total PM_2.5_ concentrations, which is defined as the relative change in model simulated PM_2.5_ concentrations with vs. without anthropogenic Nr emissions. The N-share caused by anthropogenic Nr emissions contributes about 29% (range, 17–31%) to PM_2.5_ pollution in Western Europe and 12% (8.7–16%) in Eastern Europe for 2015, exceeding 50% in some parts of Western Europe. The N-shares of NH_3_ emissions are larger than those of NO_x_ emissions and close to the N-shares of total Nr emissions because the NH_3_ reductions curtail both contributions of NO_x_ and SO_2_ to SIAs formation, which is in agreement with results of Gu et al.^18^.

**Fig. 2:**
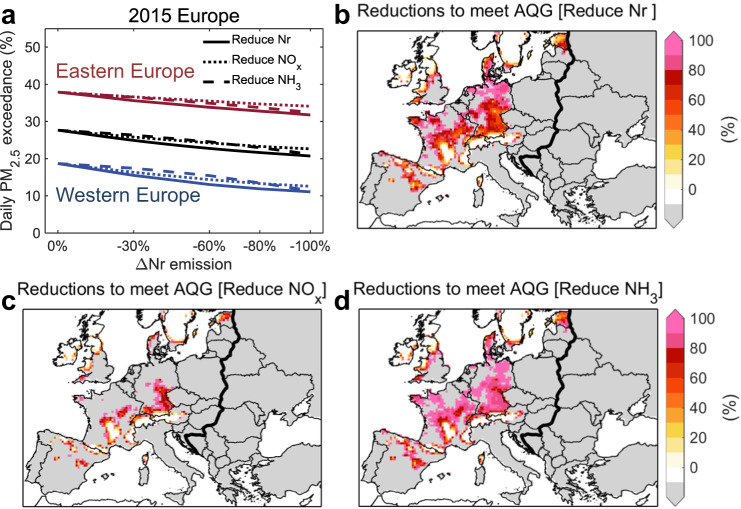
Impacts of reactive nitrogen (Nr) emission controls on the World Health Organization (WHO) air quality guideline (AQG) achievement. **a** Changes in regional mean daily WHO AQG (15 μg·m^-3^) PM_2.5_ level exceedance when European Nr (solid lines), NO_x_ (long dash lines), and NH_3_ (short dash lines) emissions are gradually decreased in 2015. **b**–**d** the reduction in Nr (**b**), NO_x_ (**c**), NH_3_ (**d**) emissions required to meet the WHO AQG for annual mean PM_2.5_ (5 μg·m^−3^). Blue, red, and black lines in (**a**) represent changes of regional mean daily PM_2.5_ exceedance in Western Europe, Eastern Europe, and all Europe, respectively. Gray areas in (**b**), (**c**), and (**d**) represent locations where even 100% Nr emission controls cannot lead to achievement.

Nr abatement would help Europe to achieve the limit set in the updated WHO guidelines for PM_2.5_ concentrations and substantially mitigate PM_2.5_-related health burdens. In 2015, only 14% of Western Europe met the PM_2.5_ annual mean <5 μg·m^−3^ (the updated WHO guideline value) and all of Eastern Europe exceeded this guideline level. Figure 2 shows that phasing out Nr emissions prompt an additional 28% of Western Europe to achieve the guideline value for annual mean PM_2.5_. NH_3_ emission controls render twice as much area in Western Europe meeting the guideline value compared to similar strengths of NO_x_ emission controls. However, annual mean PM_2.5_ concentrations in Eastern Europe cannot reach this guideline value with Nr abatement alone and need to reduce emissions of other PM_2.5_ precursors. In addition, Western Europe and Eastern Europe have 18% and 38% of all days in 2015 exceeding the guideline value for the daily average PM_2.5_, and Nr abatement cuts the daily exceedance by 41% and 16% respectively. The Global Exposure Mortality Model (GEMM) is then applied to assess PM_2.5_-related chronic health impacts (Methods). We further find setting anthropogenic Nr emissions to zero could avoid 99,000 (95% confidence interval: 92,000-106,000) PM_2.5_-related premature deaths in Europe in 2015, decreasing the annual PM_2.5_-related mortality by 29% and 6% in Western Europe and Eastern Europe, respectively (Supplementary Fig. 5).

### The efficiency of Nr emission reductions in Europe

The analyses above illustrate a larger contribution of NH_3_ emissions to PM_2.5_ concentrations, and that phasing out NH_3_ emissions leads to larger areas and more days meeting the updated WHO guideline value for PM_2.5_ air pollution compared to phasing out NO_x_. We further investigate the effectiveness of Nr emission controls under different reduction levels in sensitivity simulations with NH_3_ and NO_x_ emission reductions of 0%, 30%, 60%, 80%, and 100% over Europe in 2015 (Methods). We should note such reduction strengths extend to very drastic changes of atmospheric conditions that, while currently not seeming realistic, guide the way towards conditions compatible with the WHO guideline values and provide the information needed to devise efficient abatement.

**Fig. 3:**
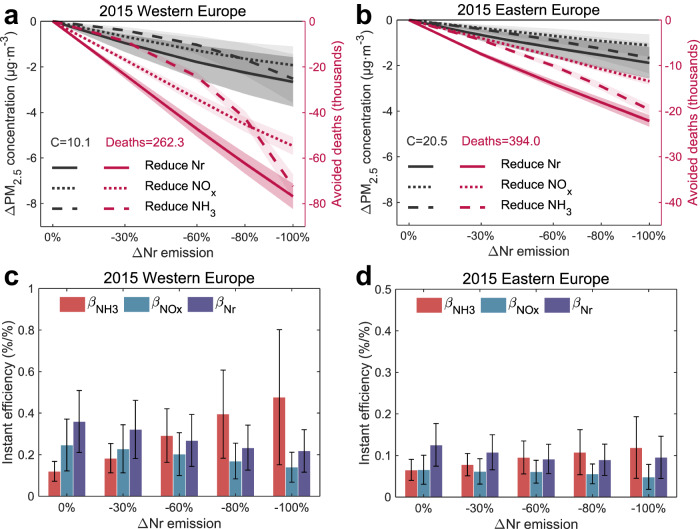
Effectiveness of reactive nitrogen (Nr) emission reductions in Europe in abating regional mean surface PM_2.5_ air pollution in 2015. **a**, **b** Reductions in Western Europe (**a**) and Eastern Europe (**b**) annual mean PM_2.5_ concentrations (black lines), and PM_2.5_-related premature deaths (red lines) when European Nr (solid lines), NO_x_ (long dash lines), and NH_3_ (short dash lines) emissions are gradually abated in 2015. **c**, **d** changes in Western Europe (**c**) and Eastern Europe (**d**) annual mean instant efficiency (refers the instant response of PM_2.5_ in percentage to 1% reduction in Nr emissions under each Nr emission scenario) associated with Nr (purple bars), NO_x_ (green bars), and NH_3_ (red bars) emission controls. The baseline simulated regional annual mean PM_2.5_ concentrations (denoted as “C”), and PM_2.5_-related premature deaths (denoted as “Deaths”) are shown in (**a**) and (**b**). Shading in (**a**) and (**b**) represent values (means ± one spatial standard deviation) of PM_2.5_ concentrations or PM_2.5_-related premature deaths. Vertical bars in (**c**) and (**d**) represent values (means ± one spatial standard deviation) of instant efficiency.

Figure 3 shows changes in regional mean PM_2.5_ concentrations and related premature deaths in Western Europe and Eastern Europe as anthropogenic Nr emissions are gradually reduced. In Western Europe, the PM_2.5_ concentrations decline non-linearly following NH_3_ emission reductions, resulting in modest PM_2.5_ changes with limited NH_3_ emission reductions, which is similar to the response found in China^26^. The regional mean PM_2.5_ concentrations in Western Europe for 2015 would decrease by 0.40 ± 0.15/1.03 ± 0.41/2.51 ± 1.06 μg·m^-3^ with 30%/60%/100% NH_3_ emission reductions in Europe. Only a deep NH_3_ abatement (up to about 80%) would yield larger total PM_2.5_ decreases in Western Europe than the same level of NO_x_ abatement. We note a difference to previous studies^33,34^ that expect higher efficiency for NH_3_ already at a much lower level of abatement, which we understand to be the result of a change in the chemical regime since these earlier studies were performed. While PM_2.5_ decreases in Eastern Europe associated with NH_3_ emission reductions tend to be more linear than those in Western Europe, the responses are similar to NO_x_ emission reductions. Notably, during summer, both regions exhibit a more linear relationship between PM_2.5_ concentrations and NH_3_ emission reductions, primarily due to the greater availability of HNO_3_ compared to other seasons (Supplementary Fig. 6). Meanwhile, we find stronger non-linear responses on PM_2.5_-related premature deaths in both regions due to the non-linear relationship between health risk and PM_2.5_ exposure; their values can be decreased by 8.7 (8.0–9.3)/24 (22–26)/72 (67–78) thousands PM_2.5_-related premature deaths in Western Europe for 2015 when NH_3_ emissions are reduced by 30%/60%/100%.

We quantify the effectiveness for PM_2.5_ reductions by calculating the instant efficiency of Nr emission controls (β, Methods). β_Nr_/β_NOx_/β_NH3_ estimates the instant response of total PM_2.5_ mass in percent for each 1% mass reduction in Nr/NO_x_/NH_3_ emissions. As shown in Fig. 3, the regional mean β_NH3_ increases rapidly while β_NOx_ slowly decreases as the level of emission reduction rises. Under the 2015 emission condition, the β_NH3_ efficiencies in Western Europe increase from 0.12 ± 0.05%/% in the base condition to 0.48 ± 0.33%/% (a factor of 4 higher) when NH_3_ emissions are reduced by 80%, while the β_NOx_ efficiencies decrease from 0.25 ± 0.13%/% to 0.14 ± 0.07%/%. The efficiencies in Eastern Europe are less than half of those in Western Europe due to the higher PM_2.5_ concentrations and lower N-share (Fig. 1), and are less sensitive to Nr emission changes.

We then apply the chemical regime metric of G ratio^25^ to explain the changes in the instant efficiency associated with NH_3_ emission controls. The G ratio denotes the ratio between free ammonia (NH_3_ and NH_4_^+^) and total nitrate (HNO_3_ + NO_3_^−^) after neutralization of H_2_SO_4_ (note, all the terms are expressed on a molar basis, Methods). The 2015 mean G ratio is found to be almost always above 1 across Europe (4.5 ± 2.4 in Western Europe and 2.8 ± 1.7 in Eastern Europe), indicating a HNO_3_-limited chemical regime and causing the SIAs formation to be more sensitive to small changes in NO_x_ emissions than those in NH_3_ emissions (Supplementary Fig. 7). This phenomenon is particularly evident in April (with a G ratio of 6.3 ± 3.2 in Western Europe and 4.2 ± 2.0 in Eastern Europe) due to the high ammonia emissions occurring in Europe during this month. In contrast, the G ratio is close to 1 in most of Eastern Europe for January, July, and October, when NH_3_ controls are slightly more efficient than NO_x_ controls (Supplementary Fig. 6). The G ratio decreases as we gradually reduce NH_3_ emissions and Europe shifts to the NH_3_-limited chemical regime, leading to NH_3_ abatement becoming increasingly effective (Supplementary Fig. 8). When we gradually reduce NO_x_ emissions, Europe remains in the HNO_3_-limited chemical regime but the β_NOx_ decreases due to decreases in oxidants (Supplementary Figs. 8 and 9). Changes in β_Nr_ depend on both β_NOx_ changes and shifts in the chemical regime. This results in a slow decrease of β_Nr_ in Western Europe and a trend of first decreasing and then increasing in Eastern Europe with deeper emission reductions.

**Fig. 4:**
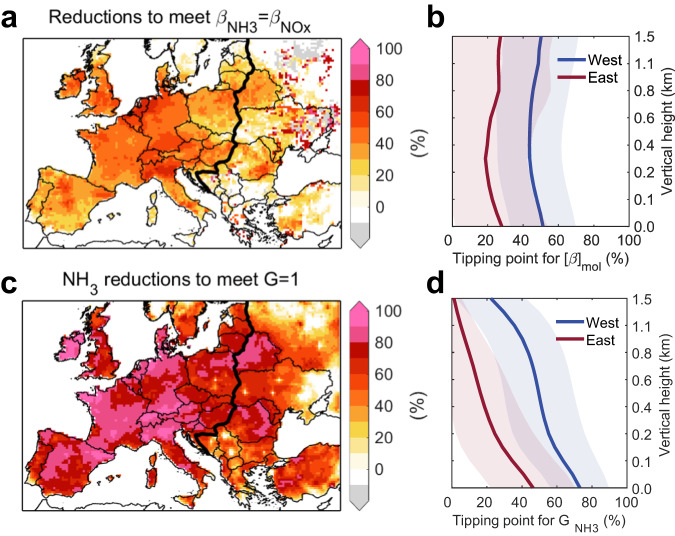
Tipping point of reactive nitrogen (Nr) emission control effectiveness targeting PM_2.5_ abatement. **a**, **c** Tipping point for surface PM_2.5_ response from NH_3_ emission reductions outweighs that from NO_x_ emission reductions identified by β_NH3_ = β_NOx_ (**a**) and the NH_3_ saturation ratio G = 1 (G, the ratio between free ammonia (NH_3_ and NH_4_^+^) and total nitrate (HNO_3_ + NO_3_^−^) after neutralization of H_2_SO_4_) (**c**). **b**, **d** Vertical profiles of Western Europe (red) and Eastern Europe (blue) mean tipping point for β_NH3_ = β_NOx_ (**b**) and G = 1 (**d**). Gray areas in (**a**), and (**c**) represent that 100% Nr emission controls here cannot achieve the tipping point. Shading in (**b**) and (**d**) represent values (means ± one spatial standard deviation) of tipping point.

A tipping point for the Nr controls can be identified where the PM_2.5_ response from NH_3_ emission reductions outweighs that from NO_x_ emission reductions, i.e., by interpolating β_NH3_−β_NOx_ or G−1 to reach zero among a series of NH_3_ and NO_x_ abatement sensitivity tests (Methods). We find that the β_NH3_−β_NOx_ tends to be positive as Nr emissions are reduced, and it has larger changes in the places with more excessive NH_3_ (Supplementary Fig. 10). Figure 4 shows the tipping point of Nr emission reductions for instant efficiencies are 36% ± 16% and 18% ± 22% in Western Europe and Eastern Europe, respectively. It indicates small mitigation for NH_3_ or NO_x_ can decrease the same PM_2.5_ concentrations in Western Europe after around 36% emission reductions in 2015. However, the G ratios are still above 1 under these abatement scenarios, and the tipping point for G ratio = 1 needs a deeper NH_3_ abatement (73% ± 16% in Western Europe and 46% ± 24% in Eastern Europe). The discrepancies between metrics of the chemical regime for SIAs formation and the effectiveness for PM_2.5_ decreases are also found in Thunis et al.^35^.

We find the first explanation for the discrepancy between the tipping point from β efficiency and that from the G ratio would be the different unit of Nr abatement, the former indicating the same PM_2.5_ decreases with per 1% mass reduction of Nr emissions and the latter indicating per unit mole reduction. This discrepancy is reduced by 36–41% when we transfer the unit of β from mass-based to molar-based. The residual discrepancy can be explained by their different definitions and applicable targets. The G ratio is based on a homogeneous air parcel at any specific moment and it loses extreme values when applied to the regional air quality model. As shown in Fig. 4, the tipping point for the G ratio falls rapidly with height while β changes steadily. Therefore, the decrease of surface PM_2.5_ depends on the chemical regime of each specific grid cell and time interval. This demonstrates that the instant efficiency is more suitable for evaluating Nr emission controls for PM_2.5_ mitigation while the chemical regime only provides a rough direction.

### The optimal pathway for Nr abatement in Europe

**Fig. 5:**
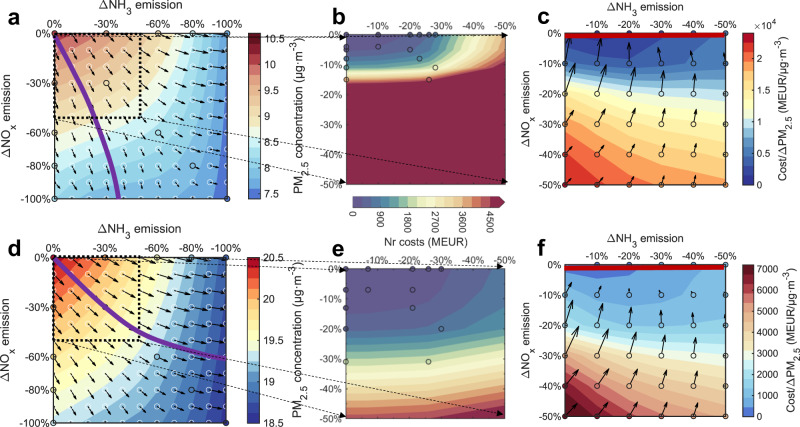
Diagnostic diagram to identify the optimal pathway of reactive nitrogen (Nr) emission controls towards effective PM_2.5_ abatement and minimal control costs. **a**, **d** the diagnostic diagram for effectiveness of regional annual mean PM_2.5_ abatement to find the optimal pathway (purple line) of Nr emission controls in Western Europe (**a**) and Eastern Europe (**d**). **b**, **e** Control costs for Nr emissions according to the NH_3_ (*x*-axis) and NO_x_ (*y*-axis) emission changes from 0 to 50% in Western Europe (**b**) and Eastern Europe (**e**). **c**, **f** the diagnostic diagram for the ratio of control costs and PM_2.5_ abatement to find the optimal pathway (red line) of Nr emission controls in Western Europe (**c**) and Eastern Europe (**f**). Black, white circles, and black arrows in (**a**) and (**d**) show 13 sets of simulated regional mean PM_2.5_ concentrations, interpolated PM_2.5_ concentrations, and their gradients in the diagnostic diagram. Black circles in (**b**) and (**e**) show control costs from five sets of feasible scenarios in the GAINS model. Black circles and arrows in (**c**) and (**f**) show the ratio of control costs and PM_2.5_ abatement at each 10% control level and their gradients in the diagnostic diagram.

Here, we develop and apply a diagnostic diagram for the effectiveness of PM_2.5_ abatement to find the optimal pathway of Nr emission controls in Europe (Methods). It visualizes the regional mean PM_2.5_ reductions as isopleths and the combined instant efficiency for PM_2.5_ abatement (the gradient) as arrows. As shown in Fig. 5, the gradient in Western Europe for the 2015 base condition tends to shift towards NO_x_, which indicates that NO_x_ emission controls would initially be most effective. By contrast, for Eastern Europe NH_3_ and NO_x_ emission controls have the similar effects in the early stage. Following the direction of gradients, we find that the optimal pathway of Nr emission controls in Western Europe entails always stronger reductions in NO_x_ than NH_3_ emissions so that the regional mean PM_2.5_ concentrations decline the fastest. This pathway approaches to reductions of ~100% NO_*x*_ emissions and 40% NH_3_ emissions inducing PM_2.5_ decreases by 2.2–2.4 μg·m^−3^, and further NH_3_ emission reductions will lead to an additional 0.4 μg·m^−3^ decrease. In contrast, the optimal pathway of Nr emission controls in Eastern Europe shall have a deeper NH_3_ abatement where ~100% NH_3_ and 60% NO_x_ emission reductions result in PM_2.5_ decreases by 1.7–1.9 μg·m^-3^.

In addition to PM_2.5_ abatement, cost considerations are also essential information for policy-making. Here we note that the optimal pathway of Nr emission controls changes uniformly in favor of NH_3_ emission reductions when we consider control costs. We quantify Nr emission abatement technologies and related costs (including investment costs, fixed and operating costs) according to the GAINS model^36–38^. Figure 5 shows the annual total costs for Nr abatement in Western Europe and Eastern Europe according to integration and interpolation among five sets of feasible emission control scenarios at the national level reported by Amann et al.^39^. Here, the costs refer to the extra annual costs incurred by the individual abatement measures to reduce Nr emissions. The abatement measures for NO_x_ emissions stem mainly from the power and industrial sectors, while those for NH_3_ are mainly from agricultural livestock farming and fertilizer use. We find NH_3_ emission controls are always cheaper than NO_x_ emission controls and Western Europe has higher costs to control Nr emissions than Eastern Europe due to the reduction policies already in place and higher levels of Nr emissions. Currently feasible emission abatement technologies in the GAINS model can reduce NO_x_ and NH_3_ emissions by 16% and 29% in Western Europe, annually costing 3.7 and 0.8 billion euros, respectively; reduce NO_x_ and NH_3_ emissions by 32% and 31% in Eastern Europe, annually costing 1.9 and 0.2 billion euros, respectively.

We further update the diagnostic diagram of cost-effectiveness using the ratio of control costs and PM_2.5_ abatement, which denotes the annual costs per unit PM_2.5_ decreases (Fig. 5). Due to the limited availability of cost data for high abatement levels, we merely extrapolate to 50% emission reductions. In Western Europe, controlling 10% (30%) NH_3_ emissions from the 2015 Base would decrease regional PM_2.5_ by 0.11 (0.40) μg m^−3^ and require implementation costs of 0.08 (1.1) billion euros. In comparison, controlling 10% (30%) NO_x_ emissions there would decrease regional PM_2.5_ by 0.23 (0.67) μg m^−3^ with implementation costs of 1.1 (12.7) billion euros. Similar cost-effective NH_3_ emission controls can be seen in Eastern Europe. The optimal pathway of cost-effective Nr emission controls follows the lowest isopleths for the cost/PM_2.5_ abatement ratio, inferring NH_3_ emission reductions only in both Western Europe and Eastern Europe. The much lower cost and increasing efficiency of NH_3_ emission control diminish the need for NO_x_ emission control.

Nr emission controls thus can help Europe towards achieving the updated WHO guideline value, reducing PM_2.5_ air pollution by 12–29% and PM_2.5_-related mortality by 6–29% in Europe in 2015. Eastern Europe (G ratios of 1–5) represents an area slightly in excess of NH_3_ where modest NO_x_ or NH_3_ emission reductions abate similar PM_2.5_ concentrations. In contrast, Western Europe (G ratios of 2–7) represents a highly NH_3_-excessive area where PM_2.5_ abatement does not become effective until NH_3_ reduction reaches above 40%. When considering control costs, the optimal pathway for halving Nr emissions clearly points towards NH_3_ management. Policy challenges specific to NH_3_ abatement have been described recently by Gu et al. ^40^. Identifying the optimal pathway for Nr emission reductions combining the effectiveness of PM_2.5_ abatement and emission control costs may also be important in many other regions over the globe, such as China, India, and the United States, where nitrogen pollution has continued to grow in recent years^41–43^. These regions are also facing heavy loads of PM_2.5_ air pollution and challenges to meet the updated WHO guideline value^7,44^. In addition to the benefits for PM_2.5_ mitigation and human health, Nr emission controls can also help reduce nitrogen deposition and surface ozone (Supplementary Fig. 9), which should be integrally considered in environmental strategies in future work.

In closing, we note that some uncertainties are associated with our analyses. First, several emission inventories based on different sectoral categories and collected by different institutions provide a considerable range of Nr emission estimates in Europe. While the estimates we use are within the range (Supplementary Table 1), the net results will be affected by the choice of Nr input. Second, the availability of NH_3_ may significantly elevate the aerosol water content and alkalinity and then enhance the production of SIAs and SOA^45,46^. The contribution of NH_3_ emissions on PM_2.5_ air pollution would thus present a lower estimate only, as here, the alkalinity is limited to the heterogeneous production of SIAs, and the SOA formation is parameterised as a multiple of OC concentrations. Furthermore, our study has not included the bidirectional exchange of NH_3_, i.e., simultaneous fluxes from and deposition to agricultural areas, which is highly uncertain and may alter the effectiveness of PM_2.5_ mitigation^47,48^.

## Methods

### Observations of air pollutants and meteorology

Daily and hourly surface chemical measurements in Europe in 2015 are obtained from the air quality database of the European Environment Agency (EEA). We use 964 background stations for PM_2.5_, which are then spatially aggregated into 565 grid cells to get a more representative evaluation of the model results. In addition, we use 27 stations for BC, 30 stations for OC, 34 stations for SIAs (Supplementary Table 2), and 21 stations for NH_3_. Of these, 26 stations containing all PM_2.5_ components are used to improve model SOA. Meteorological observations at 2072 stations in Europe in 2015 are collected from the National Climatic Data Center (NCDC), which consists of hourly 10-m wind speed (WS10), 10-m wind direction (WD10), 2-m air temperature (T2), and 2-m relative humidity (RH2). Evaluations of baseline simulated meteorology with these observations generally show good agreement (Supplementary Table 3).

### The WRF-Chem model

#### Model configuration

The Weather Research and Forecasting model coupled with Chemistry (WRF-Chem) version 4.0.3 is applied to simulate the meteorology and PM_2.5_ concentrations. The model domain includes most European countries and their surrounding regions using 150 (east-west) × 100 (south-north) grid cells at a 27-km spatial resolution. We divide the vertical atmosphere into 38 levels, with a first layer height of 10 meters above ground and a top pressure of 5000 Pa. The initial and lateral meteorological boundary conditions are based on hourly datasets from the European Centre for Medium-Range Weather Forecasts (ECMWF) Integrated Forecasting System (IFS) with a spatial resolution of 0.25°^49^. We nudge every 2-day meteorological fields with ERA5 reanalysis data to keep actual atmospheric conditions. The chemical initial and boundary conditions are driven by the CAM-Chem model output at 0.9° × 1.25° horizontal resolution^50^. Chemical processes are assessed using the gas-phase Carbon-Bond Mechanism Z mechanism (CBMZ)^51^ and the four-bin sectional Model for Simulating Aerosol Interactions and Chemistry (MOSAIC) aerosol scheme with dry diameters of 0.039–0.156, 0.156–0.625, 0.625–2.5, and 2.5–10.0 µm^52^. The SIAs formation is described in the CBMZ-MOSAIC through precursor gas oxidation (the gas-phase oxidation of SO_2_/NO_x_, the aqueous-phase oxidation of SO_2_/NO_x_ in clouds, and the hydrolysis of dinitrogen pentoxide) to form H_2_SO_4_/HNO_3_ and subsequent neutralization/condensation by/with NH_3_. The thermodynamics and phase equilibrium of SIAs are simulated by the Multicomponent Taylor Expansion Method (MTEM) and a computationally efficient Multicomponent Equilibrium Solver for Aerosols (MESA) in the thermodynamic module of MOSAIC. The gas-particle equilibrium of semi-volatile components (e.g. ammonium nitrate) is determined by the Adaptive Step Time-Split Euler Method in the gas-particle partitioning module of MOSAIC^52^. We add the heterogeneous sulfate formation reactions on particle surfaces based on Chen et al.^53^ to improve SIAs simulation. The Rapid Radiative Transfer Model for GCMs (RRTGM) scheme is used to parameterize shortwave and longwave radiation transfer^54^. Other physical parameterizations are the same as those used by Liu et al.^26^.

#### Model improvement

Our model will underestimate PM_2.5_ concentrations as the chemical mechanisms do not consider online secondary organic aerosol (SOA) formation due to its high uncertainty. Here we use a multiple of the OC concentrations to make up the SOA component by comparing simulated PM_2.5_ components to the observations (Supplementary Fig. 2). SOA concentrations are characterized as three times that of OC in summer and two times in other months^55^. This SOA assumption has little effect on our results because SIA and SOA chemistry are decoupled in the model. In addition, to account for OC underestimates, we increase the OC concentrations in Eastern and Central European countries by two and five times, respectively.

#### Model emissions

Anthropogenic emissions in 2015 use the monthly estimates from the Evaluating the Climate and Air Quality Impacts of Short-Lived Pollutants (ECLIPSE) Project at 0.1° × 0.1° spatial resolution for Europe, deriving from the GAINS (Greenhouse gas and Air pollution Interactions and Synergies) model and the Emissions Database for Global Atmospheric Research (EDGARv5.0) inventory at 0.1° × 0.1° spatial resolution for regions outside Europe (https://edgar.jrc.ec.europa.eu/dataset_ap50). We further use sector-specific diurnal weighting profiles for the anthropogenic emissions from power, industry, residential, transportation, and agriculture sectors as model hourly emission inputs (Supplementary Table 4). ECLIPSE estimates of anthropogenic NO_2_ and NH_3_ emissions over Europe in 2015 are 3.7 Tg N and 4.4 Tg N, respectively, which are consistent with current anthropogenic emission inventories (Supplementary Table 1). NO_x_ emissions have high values in the Netherlands, Belgium, and other European urban areas. While the high values of NH_3_ emissions are in the Netherlands, northern Germany, western France, and northern Italy (Supplementary Fig. 1). Biomass burning emissions adopt the Fire Inventory from the NCAR^56^. Biogenic emissions are estimated online using the Model of Emissions of Gases and Aerosols from Nature (MEGAN)^57^, except for the soil NO_x_ emissions that are from the GEOS-Chem model (http://geoschemdata.wustl.edu/ExtData/HEMCO/OFFLINE_SOILNOX/).

#### Model sensitivity simulations

We conduct a Base simulation in 2015 and a series of sensitivity simulations to examine the impacts of Nr emission reductions on PM_2.5_ air quality in Europe. First, the baseline simulation (denoted as Base) incorporates the 2015 emissions described above that have been assessed using observations. Second, a group of sensitivity simulations (denoted as S1R*N*, *N* = 30, 60, 80, and 100) reduces anthropogenic Nr emissions (both NO_x_ and NH_3_ emissions) over Europe by 30%, 60%, 80%, and 100%, respectively. The differences in PM_2.5_ concentrations between Base and S1R*N* can estimate the effects of Nr emission reductions. Third, a group of sensitivity simulations (denoted as S2R*N*, *N* = 30, 60, 80, and 100), similar to S1RN, but only reduces NO_x_ emissions. Fourth, another group of sensitivity simulations (denoted as S3R*N*, *N* = 30, 60, 80, and 100), similar to S1RN, but only reduces NH_3_ emissions. The comparison of PM_2.5_ changes between S2RN and S3RN then quantifies the respective effectiveness of NO_x_ and NH_3_ abatement. For all simulations, typical months for the four seasons (January, April, July, and October) after a 3-day spin-up for initialization are simulated to represent yearly results due to limited computing resources.

### Health-impact of Nr emission on PM_2.5_

We assess the PM_2.5_-related chronic health impacts through the Global Exposure Mortality Model (GEMM)^58^. It develops a PM_2.5_-mortality hazard ratio function according to cohort studies of worldwide outdoor air pollution, and has been widely used in recent studies^59,60^. This concentration-response function-based method focuses on total PM_2.5_ mass without assessing individual PM_2.5_ components for which evidence is limited^9^, corresponding with the GBD study^1^. The total health burden of long-term PM_2.5_ exposure is attributed to noncommunicable diseases and lower respiratory infections (NCD and LRI). PM_2.5_-related premature deaths (ΔMort) for adults (≥25 years) with age groups in 5-year intervals from 25 to greater than 85 are calculated by the following formulas:

1 $$R{R}_{i}(c)=\exp (\theta \times \,\log (\frac{z}{\alpha+1})/(1+\exp (-\frac{z - \mu }{\upsilon }))),\, z=\,{\max }(0,\, c - 2.4)$$

2 $$\varDelta Mort=\mathop{\sum }\limits_{i=1}^{12}Bas{e}_{i}\times Po{p}_{i}\times \frac{1}{R{R}_{i}}$$

where *RR*_*i*_ is the relative risk of NCD or LRI for age group *i* (*i* = 1, 2,…,12), which means the contribution of PM_2.5_ pollution to the baseline mortality rate; *c* is ambient annual PM_2.5_ concentrations, *exp* is the natural exponential function, and θ, α, μ and υ are parameters that determine the shape of relative risk in GEMM and are specified for each age group. The PM_2.5_ threshold is 2.4 μg·m^-3^, below which no impact occurs. *Base*_*i*_ is the baseline mortality rate of NCD or LRI for age group *i*, obtained from the Global Burden of Disease Study of 2015, and *Pop*_*i*_ is the gridded population within age group *i*, derived from the Gridded Population of the World version 4.11 (GPWv4) dataset. We further use the Monte Carlo method to provide the 95% confidence interval (CI) of deaths through ten thousand-time estimates.

### Indicators of Nr control for PM_2.5_ mitigation

Here, we apply a mass-based indicator of instant efficiency to quantify the effectiveness of Nr controls in reducing total PM_2.5_ concentrations, and then use a molar-based indicator of G ratio to explain changes in the effectiveness of PM_2.5_ mitigation. We calculate the efficiency of Nr emission controls based on the sensitivity simulations following the definition in Liu’s (2021)^26^. The instant efficiency is defined as:

3 $${{{\upbeta }}}_{{LN}}=\frac{\varDelta {[{{{{{{\rm{PM}}}}}}}_{2.5}]}_{{LN}} - \varDelta {[{{{{{{\rm{PM}}}}}}}_{2.5}]}_{{L}({N}{+}{1})}}{{[{{{{{{\rm{PM}}}}}}}_{2.5}]}_{base}}/\frac{\varDelta {[{Emi}]}_{{LN}}-\varDelta {[{Emi}]}_{{L}({N}{+}{1})}}{{[{Emi}]}_{base}},\, {N}=1$$

4 $${{{\upbeta }}}_{{LN}}=\frac{\varDelta {[{{{{{{\rm{PM}}}}}}}_{2.5}]}_{{L}({N}{-}{1})} - \varDelta {[{{{{{{\rm{PM}}}}}}}_{2.5}]}_{L({N}{+}{1})}}{{[{{{{{{\rm{PM}}}}}}}_{2.5}]}_{base}}/\frac{\varDelta {[{Emi}]}_{{L}({N}{-}{1})}-\varDelta {[{Emi}]}_{{L}({N}{+}{1})}}{{[{Emi}]}_{base}},\, {N}=2,\,3,\,\ldots,\,10$$

5 $${{{\upbeta }}}_{{LN}}=\frac{\varDelta {[{{{{{{\rm{PM}}}}}}}_{2.5}]}_{{L}({N}{-}{1})}-\varDelta {[{{{{{{\rm{PM}}}}}}}_{2.5}]}_{LN}}{{[{{{{{{\rm{PM}}}}}}}_{2.5}]}_{base}}/\frac{\varDelta {[{Emi}]}_{{L}({N}{-}{1})}-\varDelta {[{Emi}]}_{{LN}}}{{[{Emi}]}_{base}},\,{N}=11$$

where [*Emi*]_base_ is baseline Nr emissions (in unit of Tg N), [PM_2.5_]_base_ is baseline simulated total PM_2.5_ mass concentrations (in unit of μg·m^−3^), Δ[*Emi*]_*LN*_ is the mass of Nr emissions reductions for level *LN* (*L*1 = 0%, *L*2 = 10%, *L*3 = 20%, …, *L*10 = 90%, and *L*11 = 100%) relative to the baseline (in unit of Tg N), and Δ[PM_2.5_]_*LN*_ is associated total PM_2.5_ mass decreases (in unit of μg·m^-3^), denoting the instant response of total PM_2.5_ mass in percentage to 1% mass reduction in Nr emissions under each Nr emission scenario. PM_2.5_ concentrations on each 10% Nr (NO_x_ or NH_3_) emission scenario are calculated through a shape-preserving piecewise cubic spline interpolation among the 13 sets of simulated PM_2.5_ concentrations. We define the tipping point of NH_3_ emission reductions as β_NH3_−β_NOx_ = 0.

G ratio is applied to diagnose the availability of NH_3_ in the air and to analyze the chemical regime of SIAs formation^25^. We calculate the G ratio using the equations below.

6 $$G=\frac{[{{{{{{\rm{NH}}}}}}}_{3}]+[{{{{{\rm{NH}}}}}}_{4}^{+}]-2\times [{{{{{{\rm{SO}}}}}}}_{4}^{2-}]}{[{{{{{{\rm{HNO}}}}}}}_{3}]+[{{{{{\rm{NO}}}}}}_{3}^{-}]}$$

where [NH_4_+], [NH_3_], [H_2_SO_4_],[HNO_3_], and [NO_3_^−^] are the molar concentrations (in unit of µmol·m^−3^). Values of the G ratio below 1 mean that ammonia is insufficient to neutralize all H_2_SO_4_ and total nitrate, indicating an NH_3_-limited chemical regime where SIAs formation is limited by the availability of NH_3_^35^. In contrast, values of the G ratio above 1 mean that ammonia is sufficient to neutralize total nitrate, which characterizes a HNO_3_-limited chemical regime where SIAs formation is limited by the availability of HNO_3_. The G ratio of 1 means that changes (in mol units) in NH_3_ and NO_x_ emissions can result in similar PM_2.5_ changes. We note, that such diagnoses concerning SIAs formation to NH_3_ and to HNO_3_ availability are invalid for high temperature and low relative humidity as little ammonium nitrate aerosol is present^25^.

### Diagnostic diagram for Nr control pathways

The diagnostic diagram is developed for evaluating Nr control pathways targeting PM_2.5_ abatement (Fig. 5). We first calculate PM_2.5_ concentrations on each 10% Nr (NO_x_ or NH_3_) emission scenario (the white circle symbols in the diagnostic diagram) through a shape-preserving piecewise cubic spline interpolation among the 13 sets of simulated regional mean PM_2.5_ concentrations (the black circle symbols in the diagnostic diagram) to obtain PM_2.5_ isopleths. The isopleth gradients (the blue arrows in the diagnostic diagram) are then calculated as Eq. 7 to represent the effectiveness of PM_2.5_ reductions.

7 $$\nabla [{{{{{{\rm{PM}}}}}}}_{2.5}]=\frac{\partial [{{{{{{\rm{PM}}}}}}}_{2.5}]}{\partial x}\times \vec{{{{{{\bf{i}}}}}}}+\frac{\partial [{{{{{{\rm{PM}}}}}}}_{2.5}]}{\partial y}\times \vec{{{{{{\bf{j}}}}}}}={{{\upbeta }}}_{{{{{{\rm{NH3}}}}}}}\times \vec{{{{{{\bf{i}}}}}}}+{{{\upbeta }}}_{{{{{{\rm{NOx}}}}}}}\times \vec{{{{{{\bf{j}}}}}}}$$

Here *x* and *y* axes represent the strengths of the NH_3_ and NO_x_ emission controls, respectively. **i** and **j** are unit vectors in the *x*-direction and *y*-direction, respectively. ∂PM_2.5_/∂*x* is the gradient in the *x*-direction showing instant efficiency from NH_3_ controls. ∂PM_2.5_/∂y is the gradient in the *y*-direction showing instant efficiency from NO_x_ controls. The effectiveness diagnostic diagram can illustrate the total PM_2.5_ declines by isopleths, the combined instant efficiency on PM_2.5_ abatement by the size of arrows, and the relative efficiency between NH_3_ and NO_x_ by the direction of arrows. Therefore, the optimal pathway for effective PM_2.5_ declines can be found by following the isopleth gradients.

## Supplementary information

Supplementary Information

## Data Availability

Surface chemical measurements in Europe are obtained from the air quality database of the European Environment Agency (EEA, https://www.eea.europa.eu/data-and-maps/data/aqereporting-9). Meteorological observations are available from the National Climatic Data Center (NCDC, https://ncdc.noaa.gov/isd/data-access). Gridded population and demographic characteristics are derived from the Gridded Population of the World version 4.11 (GPWv4) dataset (10.7927/H4PN93PB, 10.7927/H46M34XX). The baseline mortality rate of noncommunicable diseases and lower respiratory infections are obtained from the Global Burden of Disease Study (https://vizhub.healthdata.org/gbd-results/). The anthropogenic emission inventory and emission control costs are available from the corresponding author on request. The data and modeling outputs generated in this study have been deposited at 10.5281/zenodo.7934101^61^ and are openly accessible.

## Code availability

The codes of WRF-Chem version 4.0.3 are available at https://github.com/wrf-model/WRF/releases/tag/v4.0.3. Codes for calculations and data processing are written in MATLAB and are available from the corresponding author upon request.
