# Supplementary material for: Optimal reactive nitrogen control pathways identified for cost-effective PM2.5 mitigation in Europe
Source: Nat Commun. 2023 Jul 17;14:4246. doi: 10.1038/s41467-023-39900-9 (PMC10352319; doi:10.1038/s41467-023-39900-9)
Supplement: Supplementary file 1 — Supplementary Information [file 41467_2023_39900_MOESM1_ESM.pdf]

Supplementary Information for

Optimal reactive nitrogen control pathways identified for cost-effective $PM_{2.5}$ mitigation in Europe

Zehui Liu[1,2], Harald E. Rieder[3], Christian Schmidt[3], Monika Mayer[3], Yixin Guo[1,2], Wilfried Winiwarter[2,4,*], Lin Zhang[1,*]

[1] Laboratory for Climate and Ocean-Atmosphere Studies, Department of Atmospheric and Oceanic Sciences, School of Physics, Peking University, Beijing 100871, China

[2] International Institute for Applied Systems Analysis (IIASA), A-2361 Laxenburg, Austria

[3] Institute of Meteorology and Climatology, Department of Water, Atmosphere and Environment, University of Natural Resources and Life Sciences (BOKU), A-1180 Vienna, Austria

[4] Institute of Environmental Engineering, University of Zielona Góra, PL 65-417 Zielona Góra, Poland

*Corresponding authors. Email: winiwart@iiasa.ac.at; zhanglg@pku.edu.cn

This PDF file includes:

Additional information on uncertainty analysis

Supplementary Figures S1 to S10

Supplementary Tables S1 to S4

Supplementary References *(1-11)*

Additional information on uncertainty analysis.

Uncertainties in anthropogenic emissions of SIAs precursors in Europe

Supplementary Table 1 shows 2015 total NO_x (in the form of NO_2), NH_3, and SO_2 emissions in Europe from three global emission inventories (EDGAR[1], CAMS[2], and CEDS[3]) and three regional emission inventories (ECLIPSE, TNO[4], and EMEP[5]). These inventories are almost at same spatial resolution (0.1°×0.1°, except for CDES is 0.5°×0.5°) and geographical extent (European continent). While differences remain in total emission estimates and detailed spatial distribution due to different standards, methods and categories[6]. For example, the emission inventories compiled directly by governments usually have a low estimate (i.e. EMEP and TNO). The estimate ranges of total NO_x, NH_3, and SO_2 emissions in Europe for 2015 are 7.0-12.5 Tg, 5.3-7.9 Tg, and 7.0-8.8 Tg, and the relative standard deviations are 24%, 17%, and 12%, respectively. These emission deviations slightly alter the effectiveness of Nr emission controls on $PM_{2.5}$ abatement, because the instant efficiency of Nr emission reductions changes slightly in the early stage (Fig. 3).

Uncertainties in air quality modeling

Our study has several limitations in the $PM_{2.5}$ model simulations. First, the availability of NH_3 significantly modulates liquid aerosol pH (increases the aerosol water content and alkalinity) and then enhances heterogenous production of secondary aerosols[7,8]. While the Model for Simulating Aerosol Interactions and Chemistry (MOSAIC) aerosol scheme in this study does not consider the heterogeneous sulfate formation on particle surface and the SOA formation. We parameterized the former based on Chen et al. (2016)[9] with a fixed liquid aerosol pH and considered SOA as a multiple of OC concentrations, which would present a lower response of $PM_{2.5}$ changes on NH_3 emission controls. Second, the bidirectional exchange of NH_3 fluxes alters the availability of surface NH_3[10,11]. However, our study ignored this process due to the high uncertainty and treated emission and deposition as separate processes, which may alter the effectiveness of NH_3 emission controls on $PM_{2.5}$ abatement. Third, the feedback between the meteorological fields and chemical fields further changes $PM_{2.5}$ formation. We nudged the meteorological conditions in WRF-Chem every two days using the FNL Analysis data to keep the simulated meteorological conditions in accordance with the actual conditions, which also hinder the response of $PM_{2.5}$ pollution on real emission reductions. Furthermore, the synergistic control of other $PM_{2.5}$ precursors (such as SO_2, volatile organic compounds, and primary aerosols) will also help Europe achieve the WHO AQG, affecting the effectiveness of $PM_{2.5}$ mitigation, which will be considered in the future study.

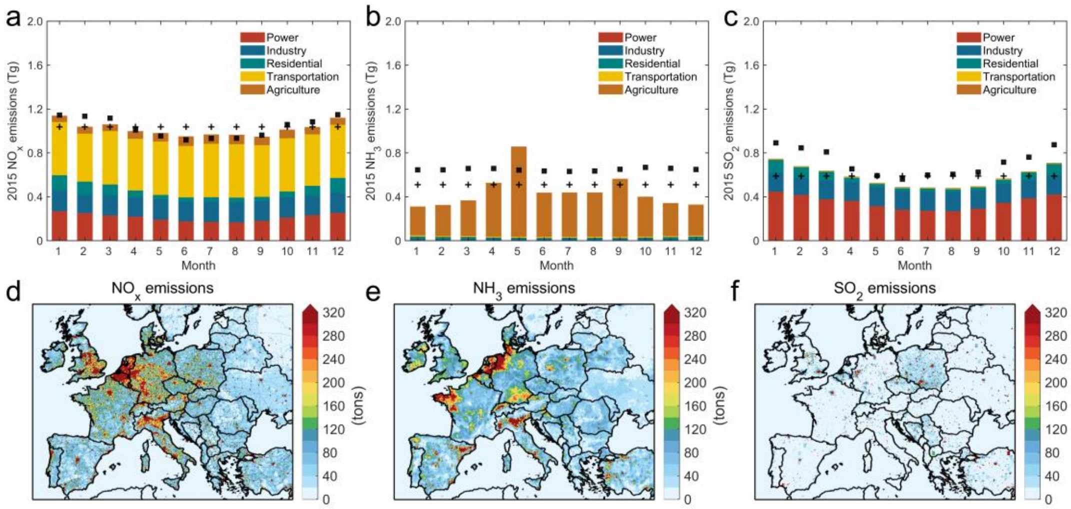

Supplementary Fig. 1 | Anthropogenic NO_x, NH_3, SO_2 emissions over Europe in 2015. a, **b**, **c**, the seasonal variation of anthropogenic NO_x (**a**), NH_3 (**b**), and SO_2 (**c**) emissions. **d**, **e**, **f**, the spatial distribution of anthropogenic NO_x (**d**), NH_3 (**e**), and SO_2 (**f**) emissions. Squares and plus signs in **a-c** represent monthly total emissions from EDGAR and EMEP, respectively.

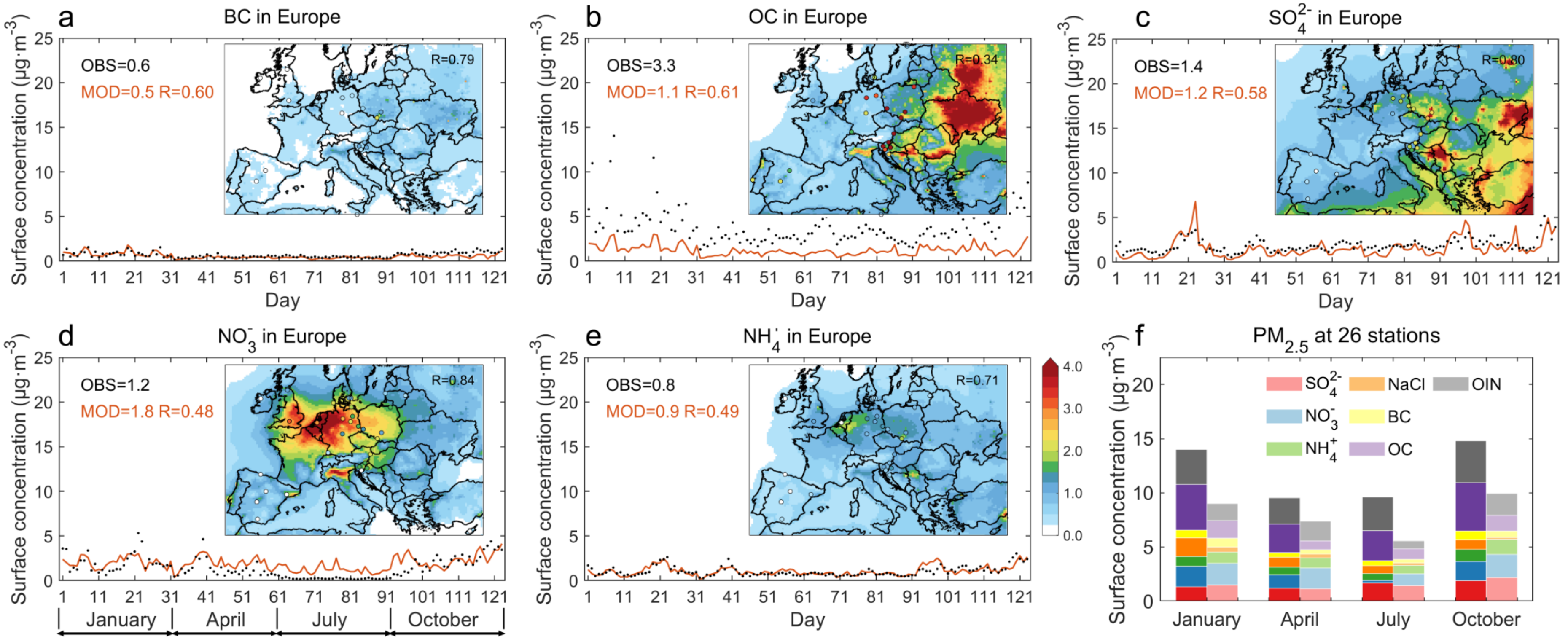

Supplementary Fig. 2 | Comparisons between observed and WRF-Chem baseline simulated $PM_{2.5}$ components. a, **b**, **c**, **d**, **e**, daily time series and spatial distribution of observed (black dots and circles) and WRF-Chem Base simulated (red lines and contours) $PM_{2.5}$ components, including BC (**a**), OC (**b**), sulfate (**c**), nitrate (**d**), and ammonium (**e**) concentrations in Europe in 2015. Mean observed values (black), corresponding model results (orange and red), and their correlation coefficients are shown as inset. **f**, comparison between observed (dark bars) and WRF-Chem Base simulated (light bars) mean $PM_{2.5}$ components at 26 stations. Mean observed values (OBS), and corresponding base simulated results (MOD), and their correlation coefficients (temporally and spatially R) are shown as inset.

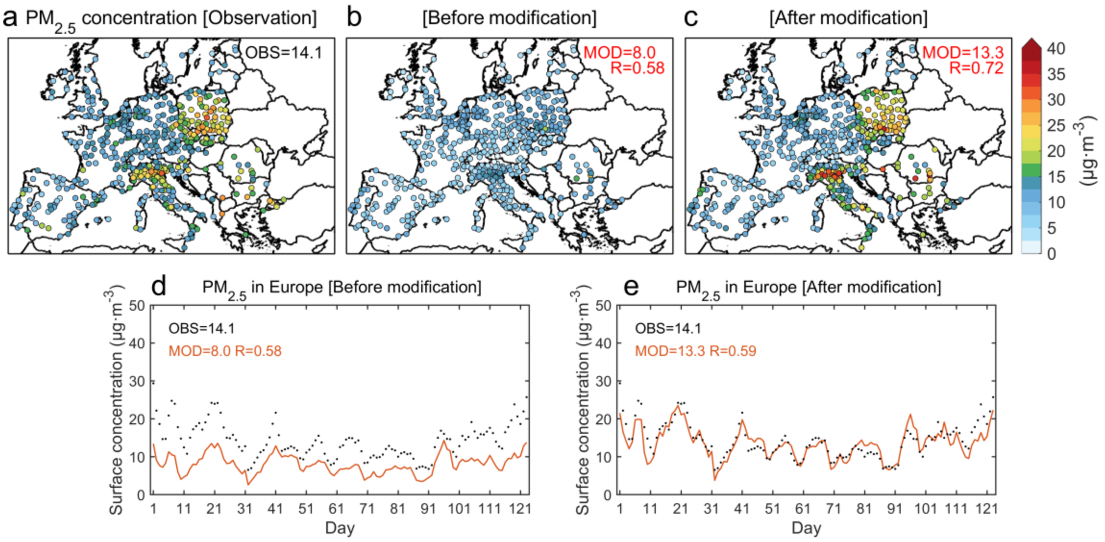

Supplementary Fig. 3 | Comparisons between observations and WRF-Chem baseline simulations before and after model modifications. a, **b**, **c**, the spatial distribution of annual mean surface $PM_{2.5}$ concentrations in Europe. **d**, **e**, time series of daily observed (black dots) and simulated (red lines) mean $PM_{2.5}$ concentrations at European stations. Mean observed values (OBS), and corresponding base simulated results (MOD), and their correlation coefficients (temporally and spatially R) are shown as inset.

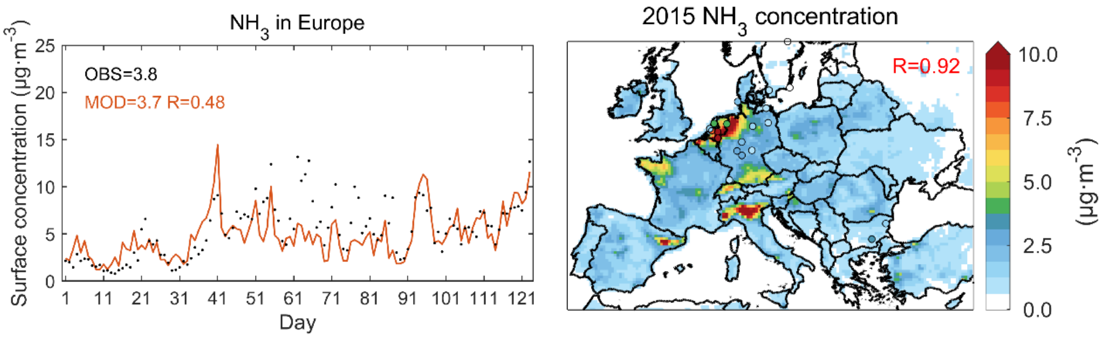

Supplementary Fig. 4 | Comparisons between observed and WRF-Chem baseline simulated surface NH_3 concentrations. Mean observed values (OBS), and corresponding base simulated results (MOD), and their correlation coefficients (temporally and spatially R) are shown as inset.

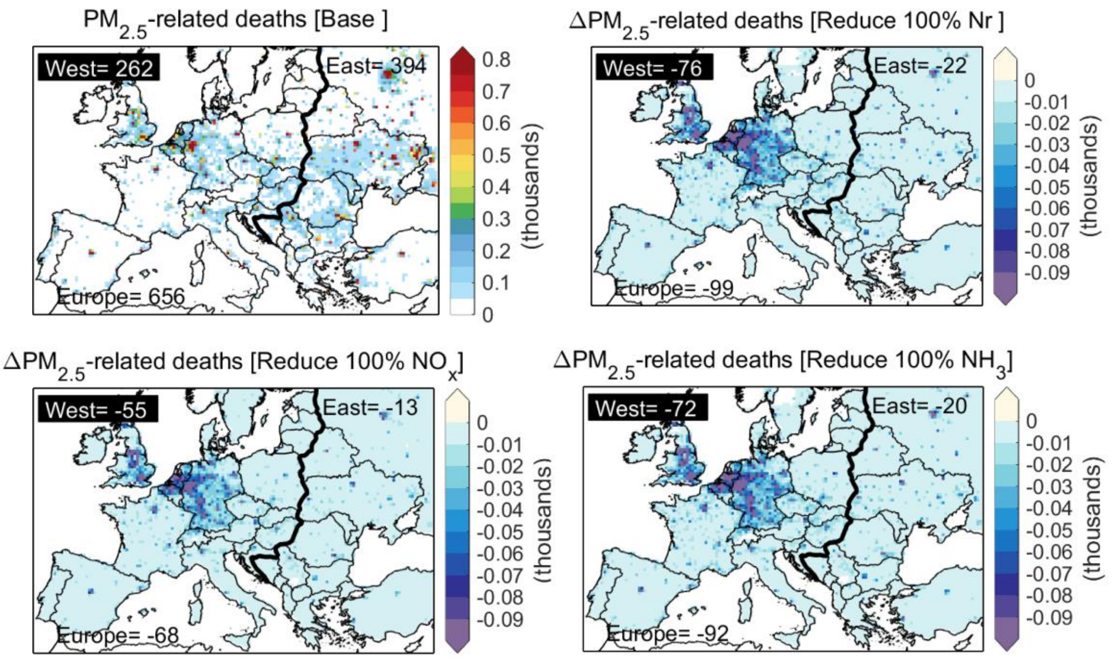

Supplementary Fig. 5 | Impacts of reactive nitrogen (Nr) emission controls on $PM_{2.5}$-related health burden. The regional total changes in Western Europe (denoted as West), Eastern Europe (denoted as East), and all Europe (denoted as Europe) are shown as inset

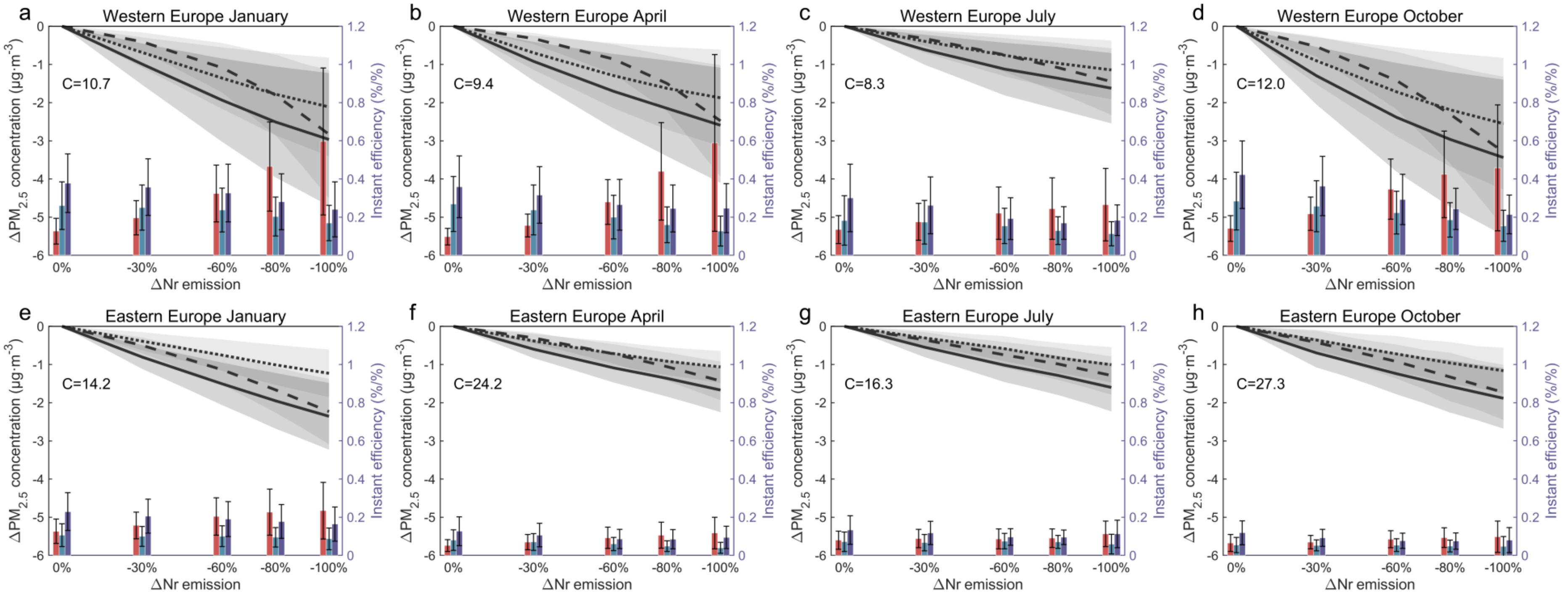

Supplementary Fig. 6 | Effectiveness of reactive nitrogen (Nr) emission reductions in Europe in abating regional surface $PM_{2.5}$ air pollution for 2015 January, April, July, and October. Reductions in Western Europe (**a-d**) and Eastern Europe (**e-h**) monthly mean $PM_{2.5}$ concentrations (black lines), and changes in monthly instant efficiency associated with Nr (purple bars), NO_x (green bars), and NH_3 (red bars) emission controls. The baseline simulated regional monthly mean $PM_{2.5}$ concentrations (denoted as "C") are shown as inset. Shading in **a-h** represent values (means ± one spatial standard deviation) of $PM_{2.5}$ concentrations or $PM_{2.5}$-related premature deaths. Vertical bars in **a-h** represent values (means ± one spatial standard deviation) of instant efficiency.

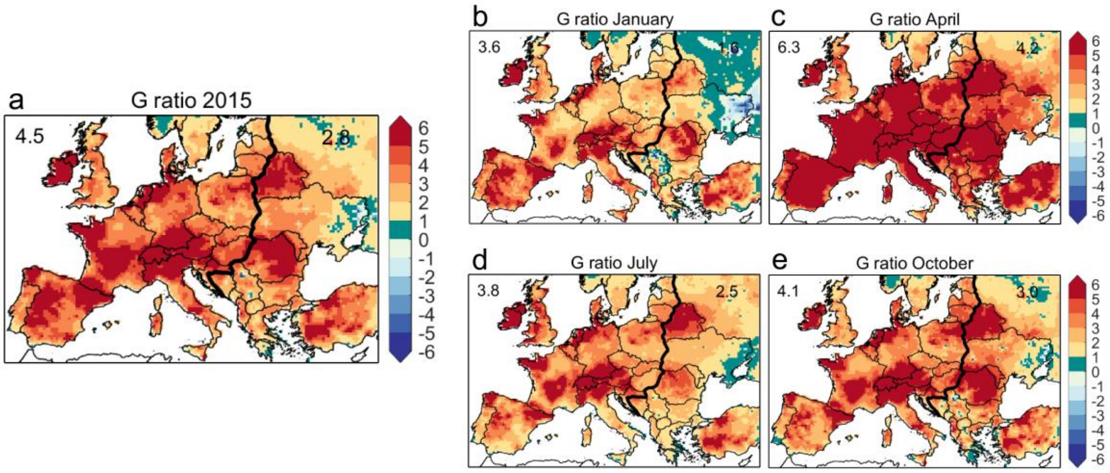

Supplementary Fig. 7 | The spatial distribution of the chemical regime (G ratio) in Europe in 2015. **a**, the annual mean G ratio in 2015. **b**, **c**, **d**, **e**, seasonal variations in the G ratio. The regional mean values in Western Europe (left values), Eastern Europe (right values) are shown as inset.

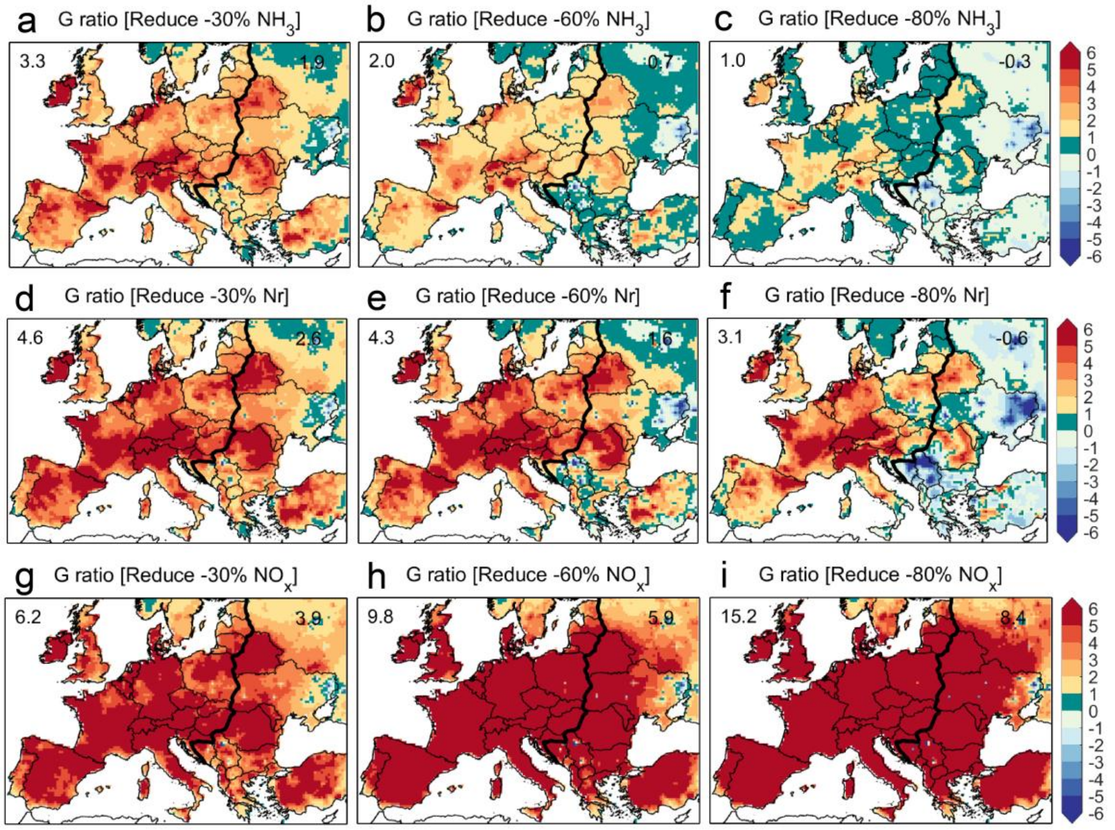

Supplementary Fig. 8 | Changes of G ratio on reactive nitrogen (Nr) emission reductions in Europe in 2015. **a**, **b**, **c**, changes of G ratio due to 30%, 60% and 80% NH_3 emission reductions. **d**, **e**, **f**, changes of G ratio due to 30%, 60% and 80% NH_3 and NO_x emission reductions. **g**, **h**, **i**, changes of G ratio due to 30%, 60% and 80% NO_x emission reductions. The regional mean values in Western Europe (left values), Eastern Europe (right values) are shown as inset.

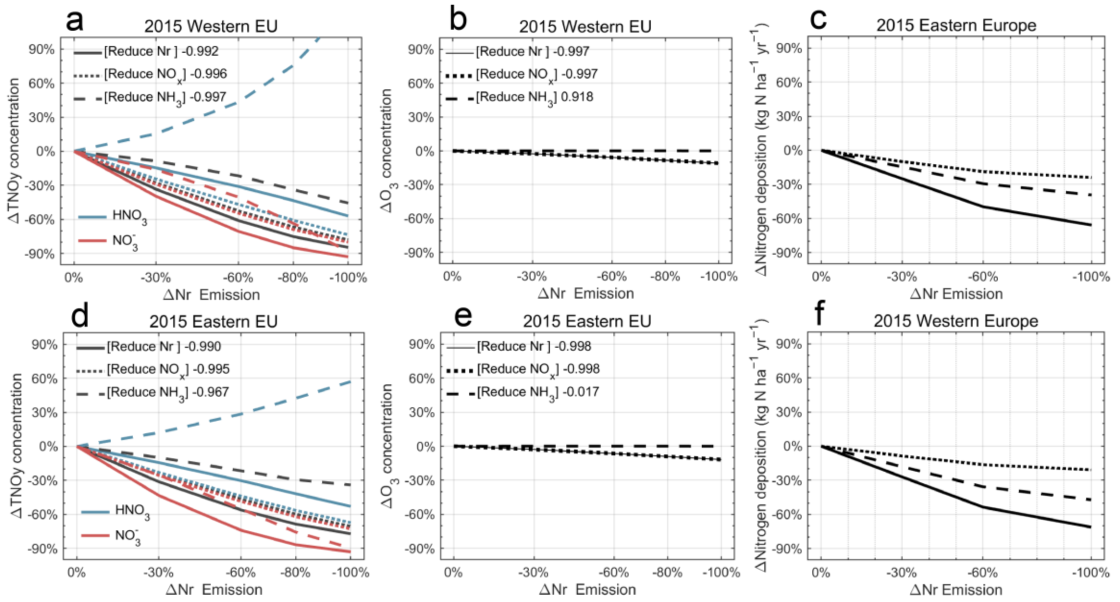

Supplementary Fig. 9 | The impacts of reactive nitrogen (Nr) emission controls on nitrate and oxidants in Europe in 2015. Reductions in Western Europe (**a-c**) and Eastern Europe (**d-e**) surface annual mean total nitrate (black lines), HNO_3 (blue lines), NO_3^- (red lines),O_3 concentrations (**b**, **e**), and nitrogen deposition (**c**, **f**) when European Nr (solid lines), NO_x (long dash lines), and NH_3 (short dash lines) emissions are gradually abated in 2015. The linear regression coefficients are shown as inset.

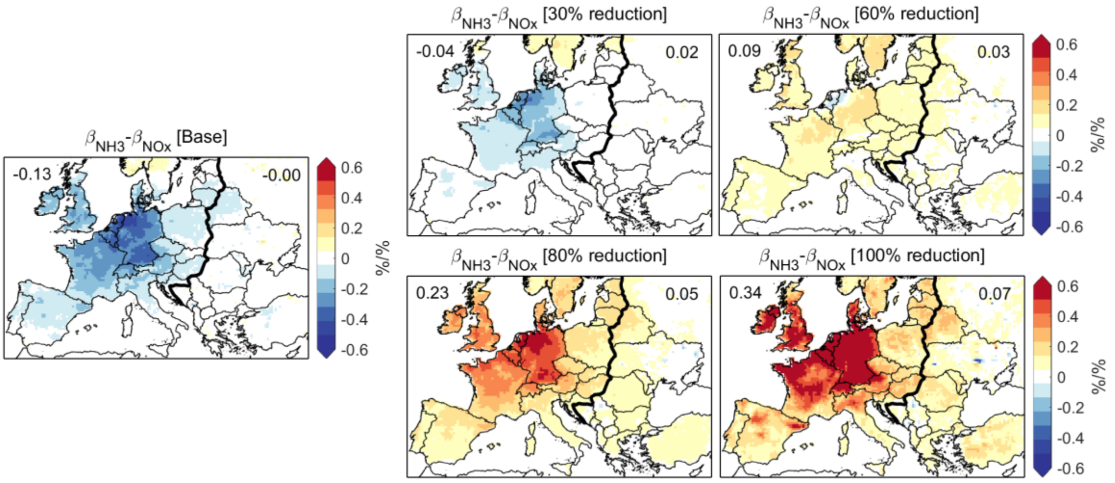

Supplementary Fig. 10 | Changes of β_{NH3}-β_{NOx} on 0%, 30%, 60%, 80%, and 100% reactive nitrogen (Nr, NH_3 or NO_x) emission reductions in Europe in 2015. The regional mean values in Western Europe (left values), Eastern Europe (right values) are shown as inset.

Supplementary Table 1 | Anthropogenic emission estimates in Europe for 2015.

Emissions (Tg N/Tg S)	ECLIPSE (this study)	EDGARv5[1]	CAMSv5.3[2]	TNO[4]	EMEP[5]	CEDS[3]
NO_x	3.7	3.8	2.3	2.1	3.5	3.7
NH_3	4.4	6.5	6.3	4.4	5.0	5.3
SO_2	4.7	5.7	5.8	4.7	4.7	5.9

EDGARv5 for 2015 is available at https://edgar.jrc.ec.europa.eu/dataset_ap50.
CAMSv5.3 for 2015 is available at https://permalink.aeris-data.fr/CAMS-GLOB-ANT.
TNO for 2015 is available at https://permalink.aeris-data.fr/CAMS-REG-AP.
EMEP for 2015 is available at https://www.ceip.at/the-emep-grid/gridded-emissions.
CEDS for 2015 is available at https://data.pnnl.gov/dataset/CEDS-4-21-21.

Supplementary Table 2 | Evaluation of WRF-Chem baseline simulated SIAs against observations from EEA over Europe in 2015.

Country	Number of sites	Sulfate				Nitrate				Ammonium			
		OBS	Base	RMSE	R	OBS	Base	RMSE	R	OBS	Base	RMSE	R
Austria*	1	2.02	0.90	1.35	-0.14	2.16	1.80	1.57	0.41	1.06	0.81	0.60	0.24
Cypern	1	3.30	3.34	1.95	0.66	0.16	0.42	0.58	0.38	1.07	1.16	0.64	0.67
Germany	9	1.61	1.31	1.19	0.72	1.76	2.57	2.09	0.54	1.06	1.19	0.85	0.67
Denmark*	2	0.75	0.43	0.35	0.93	0.90	1.05	0.30	0.92	0.47	0.43	0.07	0.99
Spain	5	0.37	0.94	1.01	0.47	0.69	0.88	1.06	0.43	0.25	0.53	0.43	0.23
United Kingdom	2	1.07	0.58	0.75	0.72	2.10	1.90	2.07	0.83	0.87	0.71	0.71	0.84
Croatia	2	1.68	1.40	1.70	0.40	0.97	1.49	1.66	0.43	1.10	0.92	0.94	0.47
Ireland*	1	0.28	0.48	0.55	0.75	0.20	1.50	2.60	0.63	0.19	0.59	1.04	-0.05
Lithuania*	1	1.29	0.56	1.01	-0.03	0.40	0.85	0.85	-0.13	0.40	0.43	0.41	-0.31
Latvia*	1	1.32	0.87	0.86	0.37	0.53	1.08	1.17	-0.10	0.52	0.60	0.52	0.27
Netherlands	1	1.46	0.97	0.82	0.71	2.93	4.31	2.75	0.69	0.18	1.53	1.75	-0.29
Poland	4	2.17	1.96	2.4	0.38	1.52	2.07	1.69	0.35	0.18	1.21	0.93	0.46
Slovenia	4	1.86	1.17	1.2	0.72	1.36	1.67	2.02	0.43	0.93	0.89	0.70	0.61
Europe	34	1.44	1.21	1.25	0.58	1.24	1.80	1.66	0.48	0.81	0.91	0.73	0.49

OBS, Base, RMSE, and R refer to mean observed values, and corresponding baseline simulated results, and their root mean squared error and temporally correlation coefficients.
* Station observations are made with a rough temporal resolution (weekly or monthly) in the country.

Supplementary Table 3 | Evaluation of WRF-Chem baseline simulated meteorological fields against observations from NCDC over Europe in January April, July, and October 2015.

		Mean [Observation]	Mean [Base]	Spatial R	Temporal R
WSP10 ($m\ s^{-1}$)	Jan.	4.5	6.1	0.46	0.67
	Apr.	3.8	5.0	0.41	0.62
	Jul.	3.4	4.3	0.70	0.54
	Oct.	3.1	4.4	0.60	0.57
WSD (°)	Jan.	208.3	211.3	0.56	0.45
	Apr.	208.3	209.7	0.75	0.43
	Jul.	208.0	209.4	0.56	0.44
	Oct.	170.9	159.1	0.45	0.44
T2 (°C)	Jan.	4.7	2.3	0.60	0.38
	Apr.	9.7	8.1	0.78	0.83
	Jul.	20.4	19.6	0.90	0.87
	Oct.	10.5	9.8	0.91	0.82
RH2 (%)	Jan.	87.3	86.5	0.54	0.41
	Apr.	72.2	72.6	0.65	0.63
	Jul.	68.3	70.8	0.76	0.72
	Oct.	83.4	83.2	0.56	0.64

Supplementary Table 4 | Diurnal weighting profiles for the anthropogenic emissions from power, industry, residential, transportation, and agriculture sectors.

Hour (UTC)	0	1	2	3	4	5	6	7	8	9	10	11
Power	0.033	0.030	0.029	0.028	0.029	0.032	0.035	0.040	0.043	0.046	0.048	0.049
Industry	0.026	0.007	0.007	0.007	0.007	0.007	0.007	0.029	0.045	0.068	0.068	0.068
Residential	0.018	0.018	0.018	0.018	0.018	0.038	0.075	0.075	0.038	0.038	0.030	0.045
Transportation	0.017	0.013	0.014	0.015	0.016	0.016	0.029	0.056	0.060	0.059	0.059	0.054
Agriculture	0.021	0.019	0.019	0.018	0.019	0.021	0.029	0.033	0.047	0.058	0.070	0.078

Hour (UTC)	12	13	14	15	16	17	18	19	20	21	22	23
Power	0.049	0.050	0.050	0.050	0.050	0.049	0.048	0.047	0.047	0.044	0.040	0.035
Industry	0.068	0.068	0.068	0.068	0.068	0.066	0.063	0.037	0.037	0.037	0.037	0.037
Residential	0.045	0.038	0.030	0.030	0.038	0.075	0.075	0.075	0.075	0.054	0.018	0.018
Transportation	0.050	0.059	0.060	0.062	0.059	0.057	0.056	0.049	0.045	0.042	0.031	0.022
Agriculture	0.089	0.082	0.080	0.070	0.060	0.044	0.030	0.027	0.023	0.021	0.021	0.021

UTC refers to Coordinated Universal Time.

References

1. Crippa, M. *et al.* Forty years of improvements in European air quality: regional policy-industry interactions with global impacts. *Atmos. Chem. Phys.* **16**, 3825-3841 (2016).
2. Granier, C. *et al. The Copernicus atmosphere monitoring service global and regional emissions (April 2019 version)*, Copernicus Atmosphere Monitoring Service, (2019).
3. Smith, S. J., Zhou, Y., Kyle, P., Wang, H. & Yu, H. In *Proceedings of the 2015 International Emission Inventory Conference, San Diego, CA, USA.* 12-16.
4. Kuenen, J., Visschedijk, A., Jozwicka, M. & Denier Van Der Gon, H. TNO-MACC_II emission inventory; a multi-year (2003–2009) consistent high-resolution European emission inventory for air quality modelling. *Atmos. Chem. Phys.* **14**, 10963-10976 (2014).
5. Mareckova, K., Wankmueller, R., Moosmann, L. & Pinterits, M. *Inventory Review 2013: Review of emission data reported under the LRTAP Convention and NEC Directive: Stage 1 and 2 review, Status of gridded and LPS data, EMEP*. (EEA, Technical Report CEIP 1/2013, 2013).
6. Trombetti, M. *et al.* Spatial inter-comparison of Top-down emission inventories in European urban areas. *Atmos. Environ.* **173**, 142-156, doi:10.1016/j.atmosenv.2017.10.032 (2018).
7. Zheng, G. *et al.* Multiphase buffer theory explains contrasts in atmospheric aerosol acidity. *Science* **369**, 1374-1377, doi:10.1126/science.aba3719 (2020).
8. Lv, S. *et al.* Gas-to-Aerosol Phase Partitioning of Atmospheric Water-Soluble Organic Compounds at a Rural Site in China: An Enhancing Effect of NH3 on SOA Formation. *Environ. Sci. Technol.* **56**, 3915-3924, doi:10.1021/acs.est.1c06855 (2022).
9. Chen, D., Liu, Z., Fast, J. & Ban, J. Simulations of sulfate–nitrate–ammonium (SNA) aerosols during the extreme haze events over northern China in October 2014. *Atmos. Chem. Phys.* **16**, 10707-10724, doi:10.5194/acp-16-10707-2016 (2016).
10. Zhu, L. *et al.* Global evaluation of ammonia bidirectional exchange and livestock diurnal variation schemes. *Atmos. Chem. Phys.* **15**, 12823-12843, doi:10.5194/acp-15-12823-2015 (2015).
11. Bash, J. O., Cooter, E. J., Dennis, R. L., Walker, J. T. & Pleim, J. E. Evaluation of a regional air-quality model with bidirectional NH3 exchange coupled to an agroecosystem model. *Biogeosciences* **10**, 1635-1645, doi:10.5194/bg-10-1635-2013 (2013).
